# Interest in Rhinoplasty and Awareness of Postoperative Complications Among Female High School Students in Riyadh, Saudi Arabia

**DOI:** 10.7759/cureus.40783

**Published:** 2023-06-22

**Authors:** Azouf M Alsulaiman, Sarah Alrashid, Sara Alabdulkareem, Raneem Najjar, Abdullah Arafat, Yazeed AlGhonaim

**Affiliations:** 1 Family Medicine, King Saud Bin Abdulaziz University for Health Sciences College of Medicine, Riyadh, SAU; 2 Medical student, King Saud Bin Abdulaziz University for Health Sciences College of Medicine, Riyadh, SAU; 3 Otolaryngology, King Saud University Medical City, Riyadh, SAU; 4 Otolaryngology - Head and Neck Surgery, King Saud Bin Abdulaziz University for Health Sciences College of Medicine, Riyadh, SAU; 5 Otolaryngology, King Abdulaziz Medical City, National Guard Health Affairs, Riyadh, SAU; 6 Otolaryngology - Head and Neck Surgery, King Abdulaziz Medical City, National Guard Hospital, Riyadh, SAU

**Keywords:** riyadh, rhinoplasty, postoperative complications, saudi arabia, awareness

## Abstract

Background

Rhinoplasty and revision rhinoplasty are facial cosmetic operations that have potentially profound psychological implications for an individual.In recent years, rhinoplasty has increased internationally because of body dysmorphic disorders but also social media influence. As with any surgery, rhinoplasty carries risks, and the aim of this study was to explore female high school students' interest in rhinoplasty and their awareness about its postoperative complications in Riyadh, Saudi Arabia.

Methods

We employed a cross-sectional design for the study, which included 413 female high school students aged 14 to 17 years. Participants signed a consent form, and data were collected through an online survey from September to November 2022. A validated questionnaire tested for reliability was used. The level of knowledge and interest in rhinoplasty was compared with the socio-demographic characteristics of the female students using the chi-squared test. A p-value of 0.05 was considered statistically significant.

Results

A total of 413 female high school students responded to our survey, and 135 completed questionnaires were received. Nearly half (48.7%) of the students were happy about the current form of their nose. Among the students, 74.3% would not consider undergoing rhinoplasty, and the most common reason was satisfaction with their nose (69.4%). Those who did intend to undergo rhinoplasty (25.7%) were primarily interested in improving their appearance (74.5%). The total mean knowledge score was 6.01 (SD 3.27) out of 12 points, with 61.7% classified as having poor knowledge levels, and the rest (38.3%) were considered as having good awareness.

Conclusions

In our perception, the number of teenagers wanting rhinoplasty is increasing. Questions about their knowledge level, attitude, and perspective regarding the procedure should be important considerations for the surgeon.

## Introduction

The nose is a prominent feature of the face and is often considered a beauty landmark. Facial appearance is closely tied to the self-image, self-confidence, and self-worth of the individual. Derangements in nasal structure, whether from surgery, trauma, or natural causes, have numerous implications for the emotional well-being of the individual [1]. People who are dissatisfied with the appearance of their nose may opt to have cosmetic surgery called rhinoplasty to alter the shape or appearance of the nose while preserving or enhancing the nasal airway [2]. Rhinoplasty and revision rhinoplasty are facial cosmetic operations with profound cosmetic and psychological effects on the patient [1].

In recent years, the number of rhinoplasty operations has increased in Saudi Arabia and currently represents 30% of all cosmetic surgery procedures. This increase is in part coming from a desire to correct a dysmorphic facial feature, but the influence of beauty norms as portrayed on social media is playing a larger role in people’s decisions [3]. It has been reported that considering a cosmetic procedure is greatly influenced by spending hours viewing cosmetic surgery-related material on social media together with the depiction of what are considered ideal features [4].

There are obvious risks involved in rhinoplasty, especially reduction rhinoplasty. Although severe complications are rare, there are many short- and long-term complications that could lead to aesthetic dissatisfaction, patient disappointment, and even functional problems [5]. Complications of rhinoplasty are grouped into two categories: aesthetic, which may require revision rhinoplasty, and non-aesthetic [6], such as breathing disturbances that are reported by 70% of all revision rhinoplasty patients. Also, damage to the skin and soft tissues can cause atrophy, fibrosis, numbness, and formation of cysts from displaced mucosa or subcutaneous granulomas. Infections are rare but can be life-threatening in the case of toxic shock syndrome. The risk is higher when sinus surgery and rhinoplasty are combined [7]. Awareness of the risk from complications should be part of an individual’s rhinoplasty decision-making process.

Although the number of adolescents electing to have plastic surgery, especially rhinoplasty, is increasing, there has been little emphasis on complications and few articles in the world’s literature have analyzed their beliefs and attitudes toward them. The aim of this study was to explore female high school students' interest in rhinoplasty and their awareness about its postoperative complications in Riyadh, Saudi Arabia.

## Materials and methods

Study design, area, and settings

A cross-sectional study design was conducted, as the study only focused on the population’s interest and awareness over a specific period of time. The survey was distributed to female schools from different areas (North, South, East, and West) in Riyadh. In Saudi Arabia, education is organized into kindergarten, primary, and secondary units, and numerous institutions offer all three units in their schools. The Saudi System first has the primary stage then splits into intermediate and secondary stages. Intermediate students are 13 to 16 years old and secondary students are 17 to 19 years old [8]. The goal of the study was to assess the interest of female high school students in rhinoplasty and their awareness of its postoperative complications in Riyadh, Saudi Arabia.

Inclusion and exclusion criteria of study participants

Our study included 413 female high school students aged 14 to 17 years who responded to the survey. Males were excluded along with students younger than 14 or older than 17 years. The data were collected online from September to November 2022.

Data collection process

After signing the consent form, the participants’ data were collected through an online survey distributed via social platforms to high school girls in all areas of Riyadh, Saudi Arabia. The questionnaire was developed by Aliasghar Arabi Mianroodi and Narges Khanjani [9]. Their permission was obtained to use the validated version in this study. It has three sections. The first section includes the subjects’ demographics obtained by a series of five multiple-choice questions. Section 2 includes five multiple-choice questions about attitude toward rhinoplasty, and section 3 has 13 short-answer questions to gauge the students’ familiarity with the postoperative complications of rhinoplasty.

Data analysis

The awareness of rhinoplasty complications was assessed using a 12-item questionnaire, where a “yes” answer was coded as 1 and “no” was coded as 0. The overall knowledge score was calculated by adding the scores on all 12 items (0-12). The higher the score, the greater the understanding of the complications of rhinoplasty. A score of 7 (60%) was used as the cutoff point to decide on the level of knowledge. Students were considered as having “poor” knowledge if the score was 7 points or below. Above 7 points was considered a “good” knowledge level.

The data for categorical variables were represented by numbers and percentages, while means and standard deviations were used for continuous variables. The level of knowledge and interest in rhinoplasty were compared with the socio-demographic characteristics of the female students by using the chi-squared test. A p-value of 0.05 or below was considered statistically significant. All data analyses were performed using the Statistical Packages for Software Sciences (SPSS) Version 26 (IBM Corp., Armonk, NY).

This study was approved by King Abdullah International Medical Research Center (IRB/1268/22).

## Results

A total of 413 female high school students responded to our survey and 135 completed questionnaires were received; the rest of the questionnaires did not meet our inclusion criteria. Table 1 shows the socio-demographic characteristics of the students. The most prevalent average age was 17 years (48.9%). The majority of the students (77.5%) were born in Riyadh City, and those living in the eastern area constituted 37.8%. Regarding parents’ education, most had a bachelor’s degree or higher (father, 71.4%; mother, 66.1%). In addition, 21.1% of the students indicated that at least one of their relatives or friends had undergone rhinoplasty (Table 1).

**Table 1:** Socio-demographic characteristics of the female high school students (n=413)

Study variables	N (%)
Age group	
14 years	02 (0.50%)
15 years	59 (14.3%)
16 years	150 (36.3%)
17 years	202 (48.9%)
School area in Riyadh	
Eastern area	156 (37.8%)
Middle area	42 (10.2%)
Northern area	126 (30.5%)
Southern area	40 (09.7%)
Western area	49 (11.9%)
Place of birth	
Inside Riyadh City	320 (77.5%)
Outside Riyadh City	93 (22.5%)
Father’s education	
High school or below	118 (28.6%)
Bachelor or higher	295 (71.4%)
Mother’s education	
High school or below	140 (33.9%)
Bachelor or higher	273 (66.1%)
Number of relatives or friends who have undergone a rhinoplasty	
None	214 (51.8%)
One	87 (21.1%)
Two	55 (13.3%)
Three	24 (05.8%)
Four or more	33 (08.0%)

Table 2 illustrates the students’ attitudes toward rhinoplasty and their knowledge about rhinoplasty complications. According to our results, nearly half (48.7%) of the students were happy about the current form of their nose. The proportion of students who were not interested in rhinoplasty was 74.3%, and the most common reason was satisfaction with their nose (69.4%). Those who intended to undergo rhinoplasty comprised 25.7% of the group and the most commonly given reason was to enhance beauty (74.5%). The most preferred quality of the doctor who would perform the operation was their work ethic (91.5%). Regarding knowledge about rhinoplasty complications, students were aware that the most common complication of rhinoplasty was dissatisfaction with the new nose (70.5%), followed by headache (70.2%) and nose blockage (68%). The total mean knowledge score was 6.01 (SD 3.27) out of 12 points, with 61.7% classified as having poor knowledge levels and 38.3% considered as good (Table 2).

**Table 2:** Students’ attitude toward rhinoplasty and their knowledge about its complications (n=413)

Attitude statement	N (%)
How do you feel about your nose?	
Happy	201 (48.7%)
Not happy	83 (20.1%)
Do not care	129 (31.2%)
Do you want to do a rhinoplasty?	
Yes	106 (25.7%)
No	307 (74.3%)
Why do you want to do a rhinoplasty? (n=106)	
For beauty	79 (74.5%)
For health reason‎	11 (10.4%)
To show off	08 (07.5%)
To catch up with the mode	04 (03.8%)
Others	04 (03.8%)
What type of doctor would you like to operate on you? (n=106)	
Doctor who works well	97 (91.5%)
Doctor with good manners	06 (05.7%)
Doctor who charges less	02 (01.9%)
Doctor who has lots of patients and is busy	01 (0.90%)
Why do not you want a rhinoplasty (n=307)	
I like my nose as it is	213 (69.4%)
Religious reasons	24 (07.8%)
Plastic surgery is of no use	21 (06.8%)
Fear of operation	20 (06.5%)
Fear of side effects	14 (04.6%)
Do not have enough money	04 (01.3%)
Other reasons	11 (03.6%)
Knowledge about rhinoplasty complication	Yes (%)
Dissatisfaction with the new nose	291 (70.5%)
Headache	290 (70.2%)
Breathing disorders	281 (68.0%)
Nose blockage	270 (65.4%)
Sensitivity to strong odors	247 (59.8%)
Recurrent nosebleed	242 (58.6%)
Nasal discharge	217 (52.5%)
Need for reoperation	194 (47.0%)
Skin discoloration	162 (39.2%)
Recurrent nausea and vomiting	121 (29.3%)
Recurrent nasal mucosal irritation	120 (29.1%)
Death	49 (11.9%)
Total knowledge score (mean ± SD)	6.01 ± 3.27
Level of knowledge	
Poor	255 (61.7%)
Good	158 (38.3%)

Table 3 shows the relationship between the level of awareness and knowledge about rhinoplasty complications and students’ attitudes and socio-demographic characteristics. The p-value was calculated using the chi-squared test. We found that the socio-demographic data and attitude toward rhinoplasty did not have a significant relationship with the level of knowledge (p>0.05) (Table 3). When measuring the relationship between interest in rhinoplasty and socio-demographic variables (Table 4), it was found that an interest in rhinoplasty was significantly more common among those students with relatives or friends who underwent rhinoplasty (p<0.001) and those who were unhappy with the current shape of their nose (p<0.001). Other socio-demographic characteristics of students did not significantly influence interest in rhinoplasty (p>0.05) (Table 4).

**Table 3:** Relationship between the level of knowledge toward rhinoplasty complications in regard to their attitude and the socio-demographic characteristics of the female high school students (n=413) ^§^P-value has been calculated using the chi-squared test.

Factor	Level of knowledge		P-value^§^
	Poor (n=255), N(%)	Good (n=158), N (%)	
Age group			
14-15 years	40 (15.7%)	21 (13.3%)	0.499
16 years	96 (37.6%)	54 (34.2%)	
17 years	119 (46.7%)	83 (52.5%)	
School area in Riyadh			
Eastern area	89 (34.9%)	67 (42.4%)	0.386
Middle area	31 (12.2%)	11 (07.0%)	
Northern area	79 (31.0%)	47 (29.7%)	
Southern area	25 (09.8%)	15 (09.5%)	
Western area	31 (12.2%)	18 (11.4%)	
Place of birth			
Inside Riyadh City	190 (74.5%)	130 (82.3%)	0.066
Outside Riyadh City	65 (25.5%)	28 (17.7%)	
Father’s education			
High school or below	71 (27.8%)	47 (29.7%)	0.677
Bachelor or higher	184 (72.2%)	111 (70.3%)	
Mother’s education			
High school or below	90 (35.3%)	50 (31.6%)	0.446
Bachelor or higher	165 (64.7%)	108 (68.4%)	
Having relatives or friends undergone rhinoplasty			
Yes	119 (46.7%)	80 (50.6%)	0.433
No	136 (53.3%)	78 (49.4%)	
Perceived happiness about nose			
Happy	127 (49.8%)	74 (46.8%)	0.841
Not happy	50 (19.6%)	33 (20.9%)	
Do not care	78 (30.6%)	51 (32.3%)	
Interest in rhinoplasty			
Yes	66 (25.9%)	40 (25.3%)	0.898
No	189 (74.1%)	118 (74.7%)	

**Table 4:** Relationship between the interest in rhinoplasty in regard to the socio-demographic characteristics of the female high school students (n=413) ^§^P-value has been calculated using the chi-squared test. **Significant at p<0.05 level.

Factor	Interest in rhinoplasty		P-value^§^
	Yes (n=106),N (%)	No (n=307), N (%)	
Age group			
14-15 years	10 (09.4%)	51 (16.6%)	0.199
16 years	41 (38.7%)	109 (35.5%)	
17 years	55 (51.9%)	147 (47.9%)	
School area in Riyadh			
Eastern area	40 (37.7%)	116 (37.8%)	0.224
Middle area	09 (08.5%)	33 (10.7%)	
Northern area	34 (32.1%)	92 (30.0%)	
Southern area	15 (14.2%)	25 (08.1%)	
Western area	08 (07.5%)	41 (13.4%)	
Place of birth			
Inside Riyadh City	78 (73.6%)	242 (78.8%)	0.265
Outside Riyadh City	28 (26.4%)	65 (21.2%)	
Father’s education			
High school or below	35 (33.0%)	83 (27.0%)	0.240
Bachelor or higher	71 (67.0%)	224 (73.0%)	
Mother’s education			
High school or below	36 (34.0%)	104 (33.9%)	0.987
Bachelor or higher	70 (66.0%)	203 (66.1%)	
Having relatives or friends underwent rhinoplasty			
Yes	70 (66.0%)	129 (42.0%)	<0.001**
No	36 (34.0%)	178 (58.0%)	
Perceived happiness about nose			
Happy	13 (12.3%)	188 (61.2%)	<0.001**
Not happy	65 (61.3%)	18 (05.9%)	
Don't care	28 (26.4%)	101 (32.9%)	

## Discussion

Our results showed that almost half of the participants were happy with the shape of their nose and were not contemplating surgery. However, more than half of the female high school students in Kerman, Iran, stated that they would like to undergo rhinoplasty [10]. This observation could be the result of a number of reasons, one of which is the diversity of cultures since this has a major effect on a child's education, beliefs, thoughts, and habits. Parental education was also stated as a mediating factor because, in some studies, as parental education increased, the rate of cosmetic surgery decreased [11]. This was in contrast to our study, which showed no relationship between the level of parental education and the surgery opt-in rate.

In our study, there were a number of reasons why participants chose not to consider rhinoplasty, with the most common being that they were happy with their nose as it was, followed by religious beliefs, fear of side effects, and financial cost. However, around 25% stated that their purpose in considering rhinoplasty was to enhance their beauty, which corroborates a similar study in Kerman, Iran [10]. However, in another study conducted in California, 30% of participants said that their choice was influenced by someone who had undergone the surgery [12]. This is mirrored in our study by the fact that the number of students who had an interest in rhinoplasty was significantly higher among those with relatives or friends who underwent the procedure.

Regarding the choice of a surgeon, the participants in this study who were considering rhinoplasty implied that they preferred to be operated on by an experienced cosmetic surgeon who had performed many successful operations. That was the primary factor that mattered most to them. In contrast, a study that was conducted in Yazd, Iran, concluded that the participants preferred to be operated on by someone with a pleasant personality who had lots of patients and did not charge as much as others [11].

Over half of our sample population had less than average knowledge about the possible postoperative complications of rhinoplastic surgery. Our students knew about the risk of dissatisfaction with the new nose, as well as the common consequences of headache and sinus blockage, but they did not consider more serious complications such as the need for reoperation or the possibility of death from infection. Another study in Iran showed that their population was more worried about the risk of reoperation and possibility that the new nose would not match the face [9].

A number of studies suggested that psychological disturbances or poor general mental health can urge people toward rhinoplasty [13,14]. In fact, a Norwegian study revealed that an interest in rhinoplasty was more likely in young women presenting with body dysmorphic disorder [15]. This was also supported by a study conducted at the Shiraz University of Medical Sciences in Iran [16]. Therefore, it would seem prudent to do some form of psychological assessment before the operation is scheduled. This subject is an important one, and further studies should be conducted in Saudi Arabia to assess the psychological aspects of an interest in rhinoplasty.

This study was limited in its generalizability because it only surveyed female high school students in Riyadh City, Saudi Arabia. Additional studies need to include both sexes and a greater range of ages.

## Conclusions

In our perception, the number of teenagers wanting rhinoplasty is increasing internationally, assessing their knowledge and understanding of the procedure and their attitudes and perspectives about it should be an important part of the patient work-up for the surgeon performing the operation. Careful attention to the individual’s reasons for desiring the surgery can contribute to a more successful surgical outcome, and an understanding of the potential risks and complications can provide greater satisfaction for the patient.

## Appendices

**Table 5:** Rhinoplasty questionnaire

	Serial number																	
	Demographics of the female high school students participating in this study																	
	Age																	
	City of birth			Riyadh										Other city				
	Father’s education			Under high school diploma			High school diploma					Graduate diploma or bachelor		Masters or doctorate				
	Mother’s education			Under high school diploma			High school diploma					Graduate diploma or bachelor		Masters or doctorate				
	Number of relatives or friends whom have done a rhinoplasty			0			1					2		3	4 or more			
	Attitude toward rhinoplasty																	
	How do you feel about your nose?			Happy				Not happy						Don’t care				
	Do you want to do a rhinoplasty?			Yes					No									
	Why do you want to do a rhinoplasty?			For beauty		To show off			Insist of friends and family				To catch up with the mode	Other reasons				
What type of doctor would you like to operate on you?		Doctor who charges less			Doctor with good manners					Doctor who operates well				Doctor who has lots of patients and is busy			Others	
Why don’t you want a rhinoplasty		Fear of operation	Fear of side effects		Don’t have enough money						Plastic surgery is no use			Like my nose as it is			Other reasons	

Are you familiar with the following post-operative Complications of rhinoplasty			
Skin discoloration	Yes	No	
Breathing disorders	Yes	No	
Recurrent nosebleed	Yes	No	
Nose blockage	Yes	No	
Recurrent nasal mucosal irritation	Yes	No	
Headache	Yes	No	
Recurrent nausea and vomiting	Yes	No	
Nasal discharge	Yes	No	
Sensitivity to strong odors	Yes	No	
Death	Yes	No	
Need for reoperation	Yes	No	
Dissatisfaction with the new nose	Yes	No	
Mismatch of new nose with the rest of the face	Yes	No	
